# Seasonal Regime Shift in the Viral Communities of a Permafrost Thaw Lake

**DOI:** 10.3390/v12111204

**Published:** 2020-10-22

**Authors:** Catherine Girard, Valérie Langlois, Adrien Vigneron, Warwick F. Vincent, Alexander I. Culley

**Affiliations:** 1 Département de Biochimie, de Microbiologie et de Bio-Informatique, Université Laval, Québec, QC G1V 0A6, Canada; Catherine5_Girard@uqac.ca (C.G.); valerie.langlois.8@ulaval.ca (V.L.); 2 Centre d’études Nordiques (CEN), Université Laval, Québec, QC G1V 0A6, Canada; adrien.vigneron.1@ulaval.ca (A.V.); Warwick.Vincent@fsg.ulaval.ca (W.F.V.); 3 Institut de Biologie Intégrative et des Systèmes (IBIS), Université Laval, Québec, QC G1V 0A6, Canada; 4 Takuvik Joint International Laboratory, Université Laval, Québec, QC G1V 0A6, Canada; 5 Département de Biologie, Université Laval, Québec, QC G1V 0A6, Canada

**Keywords:** permafrost, thermokarst pond, phage diversity, seasonality, uncultured viral genomes

## Abstract

Permafrost thaw lakes including thermokarst lakes and ponds are ubiquitous features of Subarctic and Arctic landscapes and are hotspots of microbial activity. Input of terrestrial organic matter into the planktonic microbial loop of these lakes may greatly amplify global greenhouse gas emissions. This microbial loop, dominated in the summer by aerobic microorganisms including phototrophs, is radically different in the winter, when metabolic processes shift to the anaerobic degradation of organic matter. Little is known about the viruses that infect these microbes, despite evidence that viruses can control microbial populations and influence biogeochemical cycling in other systems. Here, we present the results of a metagenomics-based study of viruses in the larger than 0.22 µm fraction across two seasons (summer and winter) in a permafrost thaw lake in Subarctic Canada. We uncovered 351 viral populations (vOTUs) in the surface waters of this lake, with diversity significantly greater during the summer. We also identified and characterized several phage genomes and prophages, which were mostly present in the summer. Finally, we compared the viral community of this waterbody to other habitats and found unexpected similarities with distant bog lakes in North America.

## 1. Introduction

Permafrost covers approximately 17% of the globe’s land surface [1] and holds an estimated 1330–1580 Pg of carbon [2]. Record high ground temperatures have been measured in permafrost-areas since the 1980s, and warming is accelerating, with average temperature increases of 0.29 °C from 2007 to 2016 across the globe [3]. Permafrost thawing can result in the release of stocks of ground carbon that are metabolized by microbes and decomposed into CH_4_ and CO_2_ [2,4,5]. It has been estimated that the contribution of permafrost thawing to the production of greenhouse gases alone to the atmosphere will result in an increase of global temperatures from 0.13–0.27 °C by 2100 [2]. Carbon released from degraded permafrost is also introduced into thaw lakes that include thermokarst lakes, small freshwater ecosystems formed from the thaw of ice-rich permafrost [6]. These waterbodies are ubiquitous features of continuous and discontinuous permafrost landscapes in northern latitudes [7] and are among the most abundant types of freshwater ecosystems in the Arctic and Subarctic [8]. Thermokarst lake processes are likely to accelerate with further warming [9] and may contribute to the further release of greenhouse gases (GHG) to the atmosphere [10,11,12], particularly in the small, shallow thermokarst lakes underlain by carbon-rich sediment [13].

Although lacustrine sediments are likely the primary contributor to GHG production in thermokarst lakes [14], the water column contains diverse bacterial communities [15], including methanotrophs and methanogens [15,16,17,18,19]. In thermokarst lakes in Nunavik, Quebec, sequencing data suggest that methanogenesis in the water column is seasonal, occurring in the entire water column in the winter and in the lower, anoxic water column in the summer. A study conducted by Vigneron et al. [17] from the water column of a thermokarst lake in this region, described the microbial populations using shotgun metagenomic and 16S-based amplicon sequencing, both in the summer and winter. The authors found that prokaryotic communities exhibited pronounced seasonal variations, with methanotrophs more prevalent in the summer along with phototrophic and aerobic pathways. Meanwhile, winter microbial communities were dominated by heterotrophic bacteria, including methanogens, and presented reductive and fermentative pathways associated with the breakdown of organic matter [17]. Temperature also appears to exert significant control on these communities: an in vitro study using sediments from thaw ponds in subarctic Canada subjected to different thermal regimes showed that the production of GHG increased with increasing temperature, with significant shifts in the bacterial community structure [20]. A metanalysis of data from northern thermokarst lakes identified a strong association between the permafrost zone, water column depth, sediment type and biome and methane emissions [13]. Environmental parameters thus exert control over carbon-cycling bacteria in thaw lakes. Nevertheless, little attention has been given to control of microbial populations in these waters by viruses, which can control bacterial communities through infection [21].

Viruses are major players in aquatic ecosystem function [22,23,24], including in freshwater environments [25,26,27,28]. They catalyze the exchange of genes between their hosts [29], which can affect a community’s adaptive responses. Viruses are also important in the biogeochemical cycling of nutrients (including carbon), as they are involved in the exchange of organic matter between dissolved and particulate pools [21]. This could have important implications for the bioavailability of carbon to methanogens and emissions of GHG in thermokarst lakes. In spite of their importance in the microbial loop and their potential influence on higher trophic levels, aquatic viral populations remain largely uncharacterized [30], including in subarctic ecosystems. To our knowledge, only one study of viral populations has been conducted in thermokarst lakes [31], which focused on myoviruses and chloroviruses using targeted amplicon sequencing. Another study conducted in non-permafrost ponds in western Canada found that increasing temperatures led to increased viral counts and shifts in the relative contribution of top-down and bottom-up processes to the control of microbial communities [32]. This suggests that complex interactions between warming temperatures, viruses and their microbial hosts may occur in permafrost thaw lakes. Elsewhere in the Arctic, soil viruses have been found to be important contributors to carbon cycling along a permafrost thaw gradient and may even participate directly in the degradation of complex carbon via viral glycoside hydrolases [33].

In the context of thermokarst lakes becoming more important sources of GHG emissions with permafrost thawing, and towards an ultimate understanding of the role of viruses in the microbial metabolic processes that contribute to global warming it is critical to answer some first order questions about the viral ecology of these pervasive ecosystems. The purpose of this study was to compare the winter versus summer diversity of prophages and dsDNA viruses in a subarctic thermokarst lake using shotgun metagenomics. The cellular microbial community of this pond has already been described by Vigneron et al. [17], and our aim was to evaluate whether viral diversity and viral reproductive strategies were stable seasonally or varied over time in this extreme environment, as was demonstrated for cellular microbes [17]. We hypothesized that like their putative hosts [17], viral communities would show distinct seasonality, with little overlap in viral community composition, and that winter would favor lysogeny. Our analysis also included a comparison of viromes from this study and from related environments elsewhere, and an evaluation of environmental parameters that may drive viral diversity in thermokarst lake ecosystems.

## 2. Materials and Methods

### 2.1. Site Description

Samples were collected in the Sasapimakwananisikw (SAS) River Valley in Northern Quebec, near the village of Whapmagoostui-Kuujjuarapik (Quebec, Canada, 55°16.5′ N, 77°45.5′ W). Samples were collected in August 2015 (hereafter referred to as the summer sampling) 0.5 cm below the surface and in March 2016 (winter sampling) directly under ice cover. The valley is located in the sporadic permafrost zone of the Subarctic and contains numerous thermokarst thaw ponds and lakes. With accelerated thawing in the region [34], terrestrial (permafrost-originating) carbon is the main source of dissolved organic matter (DOM) in the SAS ponds [35].

The small thermokarst lake sampled in this study (SAS2A, 55°13.160′ N, 77°41.80′ W) is approximately 196 m^2^ in area and 2.8 m deep. A comparison of the water chemistry in the lake in summer and winter can be found in Table 1. For a full description of this site, see [17,36].

### 2.2. Sample Collection and Processing

Sample collection, nucleic acid extraction, library preparation, sequencing and assembly were done by Vigneron et al. [17], and are briefly described here. Samples were collected in triplicate as described in [17]. Briefly, water was collected using a 3 L Limnos Water sampler (LIMNOS.pl, Komorów, Poland). Prior to sampling, the Limnos bottle was washed with 10% *v*:*v* HCl (ACS grade, Sigma-Aldrich, Oakville, ON, Canada), rinsed three times with MilliQ water (18.2 MΩ.cm) and rinsed three times with site water. Summer samples were collected at a depth of 0.5 m at three points near the middle of the pond, and winter samples were collected through three holes bored in the ice (0.6 m thick at the time of sampling) near the center of the lake, immediately under the ice cover. Water from the Limnos sampler was transferred into low-density polyethylene Cubitainers™ that had been previously washed with 2% *v*:*v* Contrad™ liquid detergent, 10% *v*:*v* HCl, and rinsed with MilliQ and site water. Samples were kept in dark and cool conditions until returned to the laboratory. Approximately 300 mL of water was filtered onto 0.22 µm Sterivex™ filters (Millipore-Sigma, Burlington, MA, USA), and filters were stored at −50 °C until processing. Water chemistry data were collected as reported in reference [17], with results shown in Table 1.

DNA and RNA were extracted from Sterivex™ filters using a modified version of the Qiagen Allprep DNA/RNA Mini Kit (Qiagen, Toronto, ON, Canada) [37]. DNA was immediately stored at −20 °C. RNA was converted to cDNA with the High-Capacity cDNA Reverse Transcription kit (Applied Biosystems) and stored at −20 °C until library preparation. Libraries were prepared with the Nextera XT Library kit (Illumina) and sequenced on an Illumina NextSeq at the CGEB—Integrated Microbiome Resource at Dalhousie University (Halifax, NS, Canada). Read quality was verified with FastQC [38] and then quality-trimmed using Trimmomatic (v0.36) [39]. Paired-end reads were assembled with IDBA-UD [40], producing 2,703,036 contigs. The assemblies and reads from Vigneron et al., were used by us in the following analyses described below.

### 2.3. Identification of Viral Reads

VirSorter (v1.0.3) [41] and VirFinder (v1.1) [42] were used to identify viral contigs in the metagenomes. Contigs over 10 kb that fell into VirSorter categories 1 and 2 (viruses) as well as 4 and 5 (prophages) were kept (209 contigs). VirFinder was also trained with a model which also included eukaryotic viruses (https://github.com/jessieren/VirFinder, accessed on 14 February 2019) and contigs with a score >0.8 and a length >10 kb were also kept (275 contigs). Half the contigs identified by VirSorter were included in the VirFinder contigs (35% of all viral contigs). The final viral dataset contained 359 contigs (minimum length 10,057 bp, maximum 83,527 bp). One phi-X sequence, which was added during sequencing, was identified using BLASTn (BLAST+, v2.9.0) [43,44] using the phi-X174 genome (NCBI accession number: NC_001422.1) and removed from the final viral dataset.

Viral contigs were clustered into consensus sequences (hereafter referred to as viral populations, a proxy for taxa) at 95% over 85% of contig coverage with the ClusterGenomes (v1.1.3) software on the iVirus.us platform [45], grouping sequences sharing >95% identity over 85% of their length into a cluster, the longest sequence of which was designated as the viral operational taxonomic unit (vOTU). Raw reads were trimmed using Trimmomatic (v0.36) [39] and were mapped to these vOTUs using Bowtie2 (v2.3.4.1) [46], after being indexed with samtools (v1.8) [47]. Read2RefMapper (v.1.1.0) [33] was then used (cov_filter = 85, percent-id = 0.95) to calculate the abundance of viral taxa within each sample, normalized by vOTU sequence length, producing a vOTU table containing 351 vOTUs. For full sequence processing information, see Supplementary Table S1. Viral reads were extracted from Bowtie2 outputs using samtools (selecting reads that successfully mapped to viral-identified contigs). Coverage estimates of these viral reads were computed using Nonpareil 3 [48].

### 2.4. Diversity Analyses

Diversity analyses were performed on the vOTU table. Statistical tests were performed with R (v3.6.1.) [49], and *p*-values are reported for significant results only (α = 0.05). Unless stated otherwise, graphics were plotted with ggplot2{} (v3.2.1) [50] in R. To correct for sequencing depth bias (Supplementary Figure S1, Supplementary Table S2), we normalized the vOTU table (*n* = 351) using cumulative sum scaling (CSS) in metagenomeSeq{} [51]. This normalized vOTU table was used to calculate viral diversity. Alpha diversity was inferred using Shannon’s index (to measure evenness) in the phyloseq{} package (v1.30.0) [52] in R. The number of vOTUs identified in each sample was calculated by the number of non-zero lines in the vOTU table.

When assumptions for parametric tests were met (tested with shapiro.test() and bartlett.test() in stats{}), Student’s *t*-test in stats{} was used to compare sample means. The Mann–Whitney non-parametric test in stats{} was used when parametric assumptions were not met.

### 2.5. Genomic Analyses

Prokka (v1.13.7) [53] was used in viral mode to identify coding sequences (CDS) within the vOTUs. Circular viral genomes (*n* = 9) and prophages (*n* = 8) were identified by VirSorter and plotted as genomic maps from Prokka-generated gbf files with SnapGene^®^ software. To determine possible taxonomy and host, circular vOTUs were included in a viral proteomic tree computed with ViPTree [54] to assess relatedness to known genomes. Candidate host and viral taxonomy for prophage-identified vOTUs were explored with BLASTx. Annotation of selected genomes was performed using PHMMER (Webversion 2.41.1) [55] from Prokka-identified CDS, using “viruses (taxid: 10239)” as a taxonomic group and UniProtKB as a reference database.

### 2.6. Comparison to Other Viral Communities

To compare the SAS2A surface virome dataset to known viral genomes, vOTUs were compared to a database containing >125,000 metagenomic viral contigs [56] (available at http://portal.nersc.gov/dna/microbial/prokpubs/EarthVirome_DP/Nature_Protocols/reference_metagenomic_virus_database/, accessed on 29 May 2020) as well as a permafrost viral metagenome published by Emerson et al., (2018) [33] (GenBank accession number QGNH00000000.1) with BLASTn as described by Paez-Espino et al. (90% identity over 75% of the shortest sequence) [57], and habitats were identified from metadata in the Integrated Microbial Genomes/Virus database (IMG/VR) when available. This analysis included contigs identified by the VIRSorter and VirFinder pipeline that were between 2 kb and 10 kb in length. Contigs shorter than 10 kb cannot be conclusively identified as viral in origin [58], and thus these sequences (which we categorized as “virus-like”) were only used in the comparison with other datasets.

### 2.7. Data Availability

Raw sequences are available in the NCBI SRA database (Bioproject PRJNA515027). Viral contigs as well as raw and processed vOTUs tables are available on the ViDEL GitHub site (https://github.com/LabViDEL/SAS2A, available as of 21 October 2020).

## 3. Results and Discussion

### 3.1. Viral Yield

The low number of vOTUs identified from our metagenomes (Supplementary Table S1) is due to these reads being acquired from water filtered onto 0.22 µm Sterivex^TM^ filters that were comprised mostly of cellular genomes. By focusing on the viral component of the cellular-sized fraction, we were able to capture intracellular viruses and prophages, as well as viruses >0.22 µm in size and viruses adsorbed to larger particles including cells. This size fraction, which is typically removed when harvesting aquatic viruses during pre-filtration, allows a more direct assessment of the active (intracellular) and lysogenic (prophages) component of the viral community.

### 3.2. Contrasting Environmental Conditions and Viral Populations Across Seasons

**Figure 1:**
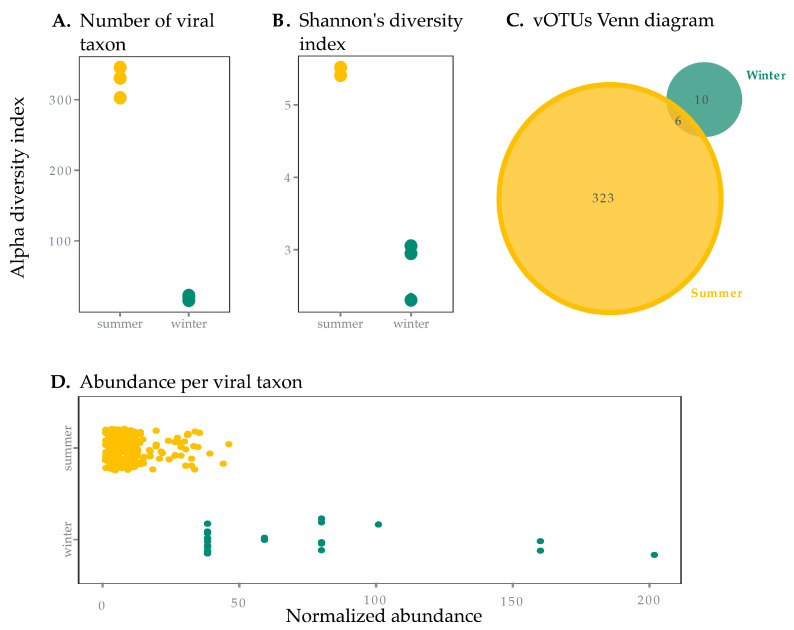
Viral sample richness (within sample diversity) and taxa abundance in summer and winter surface samples from SAS2A. Diversity results are presented using the (**A**) number of viral taxa present (presence of a viral operational taxonomic unit (vOTU)) and (**B**) the Shannon diversity index (to account for evenness). Summer samples had significantly higher diversity in terms of the number of viral taxa (**A**) but the difference between seasons was not significantly different for the Shannon Index (**B**) (*p* > 0.05). (**C**) We also present a Venn diagram for vOTUs found in summer and winter, showing there is little overlap between seasons (6 vOTUs, 1.7%). (**D**) Abundance per viral taxon is greater in the winter, with a few taxa dominating the community, supporting observations from Shannon’s diversity index (**B**).

The SAS2A thermokarst lake exhibited vastly different conditions in the summer and the winter. (Table 1). These dramatic seasonal differences were associated with contrasting viral richness between summer and winter surface samples. Of the 351 vOTUs detected in this study, the majority were found in the summer. Indeed, the number of vOTUs was over 15 times greater during the summer than during the winter (Figure 1A, *p* < 0.05). Summer samples had a higher Shannon diversity index than winter samples, suggesting lower evenness in winter samples (Figure 1B). This is shown in the dominance in abundance of a few select vOTUs in winter (Figure 1D), which were individually more abundant than the more even (and diverse) summer vOTUs. Curves showing estimates of metagenomic coverage show that the sequencing effort for winter samples was overall lower (Supplementary Figure S1)—this may be due to differences in sample collection and storage, DNA extraction and library preparation [59], and redundancy analyses conducted with Nonpareil [48] showed that winter coverage, while at least >84%, was lower than in summer (Supplementary Table S2). We thus performed normalization to correct for under-sampling of winter samples (see Section 2.4), which reduced our size factor difference between summer and winter from 73 to 3×. While it is possible that this lower coverage in winter samples (even in our normalized table) may explain a lower diversity, the 10-fold difference in the number of observed taxa between seasons seems too large to be explained by sampling bias alone and supports the idea that differences are biological in nature.

There was little overlap between the summer and winter vOTUs (Figure 1C). These results suggest a strong partitioning of viruses between seasons, concurrent with the observations in the host communities in the same lake. Indeed, amplicon sequencing of the 16S RNA gene and transcripts as well as metagenomic reads from these samples showed that aerobic and phototrophic organisms were the most abundant members of the microbial community in the summer while methanogens were more abundant in the winter [17]. Furthermore, a study of eukaryotes in the same lake (though in the summer only) showed an abundant and diverse community of phototrophs [60]. Microbial phototrophs (both eukaryotic and prokaryotic) are therefore more abundant in surface waters in the summer. The dramatic seasonal shift observed in the surface viral community, mirroring the shift observed in prokaryotes, indicates that host community composition is a key driver of viral diversity. These data also suggest that the winter column is not an important reservoir for “summer” viruses, despite conditions that favor longer residence times (minimal exposure to UV radiation and lower cell abundance and thus lower concentrations of adsorptive particles and degrading extracellular enzymes).

### 3.3. Genomic Analyses of Uncultured Viral Genomes

**Figure 2:**
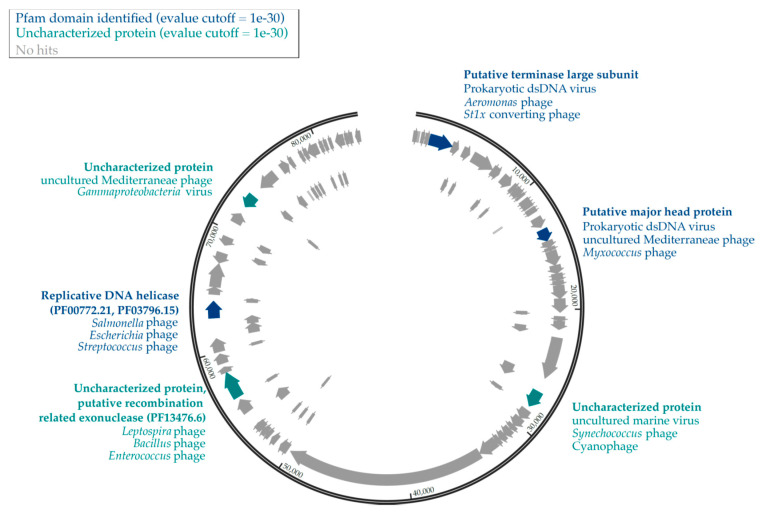
Genomic map of UViG11. Pfam-identified proteins and lineage of best hits are shown in dark blue. Uncharacterized proteins which had an e-value <1 × 10^−30^ are presented in teal, and proteins with no hits in the UniProtKB database are in gray.

Among the vOTUs identified in our dataset, we found 9 complete (circular) viral genomes, and 8 putative prophages (Table 2). All the viral genomes assembled in this study were present in the summer samples, except for one which was also present in the winter (Figure 1C, Table 2) and was the longest genome in our dataset (83,527 bp). We compared these complete genomes to the IMG/VR database, which includes cultivated viruses and Uncultivated Viral Genomes (UViGs) derived from viromes from a wide range of locations and environments [56,57]. Using relatively strict criteria (90% identity covering 75% of the shortest genome in the alignment), we were not able to identify any homologues in the database, an indicator of the apparent novelty of these thermokarst lake viruses. Protein coding sequences (CDS) were identified with Prokka, and UViGs contained between 11 and 107 CDS, with the number of CDS corresponding to the length of the genome (adjusted R^2^ = 86%, *p* < 0.0001) (Supplementary Figure S2). Meanwhile, the 8 prophages contained between 9 and 18 CDS, and varied in length from 10,785 to 29,945 bp. The vast majority of prophage CDS were annotated as hypothetical proteins (Figure 2). Our expectation was that winter conditions, featuring lower host metabolic activity and the absence of primary production would favor lysogeny, as seen in other polar environments [61]. The recovery of prophages only from summer samples was contrary to this hypothesis. However, these results may be influenced by the relatively lower sequence coverage of the winter samples, the quality of the assembly and the limits of the bioinformatic tools used to identify integrated viruses.

Using a proteomic tree computed with VipTree containing the 9 circular UViGs and 1201 reference genomes, we identified potential viral and host taxonomy (Supplementary Figure S3). Circular UViGs are suspected to infect Firmicutes, Gammaproteobacteria or Alphaproteobacteria, and putatively belong to a wide range of viral families (Table 2). Vigneron et al. found that these (putative host) groups were among the most abundant in the summer [17]. However, the validity of this approach to identifying host groups and assigning taxonomy is questionable as it is unable to account for the mosaicism of viral genomes and assumes a common origin for viruses that may have arisen independently [62]. In addition, most of the UViGs from this study have deep branches indicating a distant homology with classified viruses which is likely a reflection of the paucity of viruses in the database when compared to the true viral diversity, and the novelty of the viruses in thermokarst lakes.

We annotated the largest viral contig in our dataset (UViG11, 83,527 bp) which was identified as circular by VirSorter and contained 107 putative CDS (Figure 2). Tentative host associations through ViPTree suggested that the host was among the halobacteria (Table 2), a group of organisms that are present and active in SAS2A [19]. However, UniProtKB viral protein comparisons through PHMMER identified proteins from hosts including cyanophages but also for Proteobacteria (*Myxococcus*), Spirochaetes (*Leptospira*), Gammaproteobacteria (*Salmonella*) and Bacilli (*Enterococcus*, *Bacillus*, *Streptococcus*). Only two proteins were identified to a Pfam domain, a replicative DNA helicase (PF00772.21, PF03796.15) and a putative recombination-related exonuclease (PF13476.6).

### 3.4. Comparisons to Other Sites

We compared the viral communities found in SAS2A to publicly available viral databases, first with the IMG/VR database [57,63]. Using strict similarity criteria (90% identity over 75% of the shortest sequence in the alignment), we identified 9 high-similarity sequences (Supplementary Table S3) (out of the initial 351 vOTUs) which in many cases, were near-perfect matches (i.e., ~100% of the shortest sequence covered, alignment lengths from 6118 to 12,308 bp). The observation of identical or highly similar viral genomes or viral genes in remote, distinctly different environments is concurrent with other data [64,65,66], including in the Arctic [67]. Where available, we extracted the metadata for these genomes, and found they were all sequenced from aquatic samples in North America (IMG Genome ID listed in Supplementary Table S3A). Samples were all from freshwater habitats (either hypolimnion or epilimnion) in lakes or bogs, notably Lake Mendota, Crystal Bog Lake and Trout Bog Lake, in Wisconsin [66]. While these last two sites are in a boreal zone and not a permafrost area like SAS2A, these bog lakes nonetheless share many similarities with SAS2A. Bog lakes contain high levels of DOC [68], which gives them a similar ‘black tea’ color to thermokarst thaw lakes and ponds. These bog lakes are also stratified like SAS2A, with a highly oxygenated epilimnion and an anoxic hypolimnion. Despite their geographic distance, the high similarities between certain vOTUs identified in SAS2A summer samples to virotypes from boreal bog lakes suggest that these locations are linked. One potential means of viral exchange is via aerosols. Previous research has shown that viable viruses and their hosts can be transported long distances [69,70] and it is thus possible that there is dispersal of viruses from one of these environments to the other and vice versa. Nevertheless, a vast majority (98%) of the SAS2A vOTUs had no homology to sequences in the IMG/VR database, indicating the overall novelty of the thermokarst lake viral community.

We also identified several viral-like contigs (*n* = 66), which were obtained through our processing pipeline but did not meet the 10kb cut-off criteria (Supplementary Table S2). While these contigs are too short to be confidently identified as definitively viral, many presented high identity over moderate alignment lengths (2–7 kb) to other metagenomes from Lake Mendota, Crystal Bog Lake and Tout Bog Lake (Supplementary Table S3B), further supporting the hypothesis that our study site and these environments are linked.

As the SAS valley is heavily impacted by permafrost thawing and erosion [34], we also compared our data specifically to permafrost-associated metagenomes. Using the same approach as with the IMG/VR database comparison (see above), we compared our vOTUs to a soil viral metagenome isolated in Stordalen, Sweden from bulk soils along a thaw gradient (palsa, bog and fen) [33]. As a result that the main source of carbon in the SAS River Valley ponds is allochthonous, derived from the surrounding terrestrial permafrost environment, we expected to find similar sequences between the two datasets. While 3 of the viral-like contigs (less than 10 kb in length) from SAS2A had 98–100% identity to Stordalen metagenomes, none of our 351 vOTUs produced significant hits (Supplementary Table S4). This supports observations from the marine environment that the largest influence on viral microdiversity is local environmental drivers [71]. While these two environments (SAS2A thermokarst lake, Stordalen permafrost soil) would appear to have many characteristics in common, regional differences and low connectivity could also explain these results.

The subarctic region contains a large number of lakes and ponds, including many similar to SAS2A, that exhibit a wide range of water chemistry characteristics. These differences appear to be subsequently reflected in the diversity of prokaryotic and viral communities, which were shown to be significantly different across three waterbody types that included SAS2A in the eastern Hudson Bay region [31]. These results underline the importance of further sampling across spatial and temporal gradients to capture the full diversity of viruses in this region.

## 4. Conclusions

This study characterizes the seasonal partitioning of viral communities in a permafrost thaw lake, an environment that may be an increasingly important contributor of GHG to the atmosphere as the northern landscape continues to warm and thaw. We observed a pronounced shift in viral diversity from summer to winter that corresponded to similar changes in the cellular community. This lack of overlap in summer and winter viral taxa suggests that the water column does not serve as a reservoir for summer viruses to overwinter from year to year. We assembled several circular viral genomes and prophages, a vast majority of which had no identifiable coding regions. The presumably lytic viruses appeared to infect bacteria known to be present in the summer pond flora, while all of the prophages identified were from summer hosts, raising the possibility that lysogeny is more prevalent in this season, an observation counter to our expectation. A comparison of our data with a global database of viral sequences revealed unexpected similarities between thermokarst lakes and distant bog lake viral communities

## Figures and Tables

**Table 1:** Water chemistry of SAS2A. LD: limit of detection, TN: total nitrogen, DOC: dissolved organic carbon. Surface and bottom measurements are provided when the water column was stratified.

Season	Cover (m)	Temperature (°C)	pH	Oxygen (mg L^−1^)	CH_4_ (µM)	SO_4_ (nM)	TN (mg L^−1^)	DOC (mg L^−1^)
Summer	none	Surface: 15 Bottom: 6	6	Surface: 4.13 Bottom: <LD	Surface: 2.5 Bottom: 300	1.46	0.7	13.7
Winter	0.5 m snow 0.6 m ice	Surface: 0 Bottom: 3.5	5	<LD	200	0.5	1.2	18.3

**Table 2:** Potential viral and host taxonomy for circular viral Uncultivated Viral Genomes (UViGs) and integrated prophages identified from SAS2A metagenomes. Putative host rank was identified by ViPTree proteomic analyses for circular genomes through closely related genomes (within the same clade) (Supplementary Figure S2) and rRNA 16S extracted genes for prophages. Viral taxonomy was inferred from ViPTree. For prophages, BLASTx hits are presented for similar hits, when available. UViGs are identified as either circular (C) or prophages (P), length is given in base pairs (bp) and CDS are protein-coding features. Normalized abundance was averaged across triplicates within a season.

Genome Type	UViG	Contig ID	Length (bp)	Number of CDS	Summer Average Abundance	Winter Average Abundance	Putative Host Rank	Putative Viral Group
C	UViG3	Ga0256681_10553986	63,992	103	2.18	0	Firmicutes	Siphoviridae, Myoviridae (*Lactococcus*, *Clostridium*, *Geobacillus* phages)
C	UViG4	Ga0256681_10542696	34,037	36	7.61	0		
C	UViG5	Ga0256681_10559173	36,339	61	13.3	0	Gammaproteobacteria	Podoviridae (*Rhodoferax*, *Vibrio*, *Thalassomonas* phages)
C	UViG6	Ga0256681_11263128	62,403	87	4.54	0	Gammaproteobacteria	Podoviridae, Sophoviridae (*Pseudomonas*, *Xanthomonas* phages)
C	UViG7	Ga0256681_10579994	45,748	55	1.65	0		
C	UViG8	Ga0256681_10547172	42,853	61	8.45	0	Gammaproteobacteria	Podoviridae (*Acetinobacter*, *Aeromonas*, *Edwardsiella* phages)
C	UViG9	Ga0256681_10567549	36,908	45	6.1	0	Gammaproteobacteria	Podoviridae, Siphoviridae (*Pseudoalteromonas*, *Marinomonas*, *Escherichia* viruses)
C	UViG10	Ga0256681_10572110	37,103	56	1.63	0	Alphaproteobacteria	Siphoviridae (*Caulobacter* viruses)
C	UViG11	Ga0256681_10539383	83,527	107	5.85	2.16	Unknown	Unknown (*Natrinema*, *Haloarcula* viruses)
P	vOTU12	Ga0256681_11878260	12,576	13	0.37	0	Gammaproteobacteria	Myoviridae (*Pseudomonas*, *Stenotrophomonas*, *Xanthomonas* phage)
P	vOTU13	Ga0256681_12578680	29,945	13	9.71	0	(Bacteria, Archaea)	Unknown
P	vOTU14	Ga0256681_12584168	15,432	17	1.2	0	Unknown	Unknown
P	vOTU15	Ga0256681_12612509	13,421	11	2.46	0	(Bacteria)	Unknown
P	vOTU16	Ga0256681_10168136	15,698	15	0.89	0	Betaproteobacteria (*Polaromonas*)	Unknown
P	vOTU17	Ga0256681_10683670	11,399	18	3.47	0	Unknown	Unknown
P	vOTU18	Ga0256681_11428677	14,176	18	1.33	0	(Bacteria, Archaea)	Unknown
P	vOTU21	Ga0256681_10088446	10,785	11	9.7	0	(Bacteria, Archaea)	Unknown

## Supplementary Materials

The following are available online at https://www.mdpi.com/1999-4915/12/11/1204/s1, Figure S1: Metagenomic coverage estimates for each sample, Figure S2: Linear regression models between genome length and number of predicted CDS, Figure S3: Viral proteomic tree for circular genomes, Table S1: Processing steps from raw sequences to vOTUs, Table S2: Metagenomic coverage estimates and model coefficients, Table S3: SAS2A vOTUs similar to viral metagenomic contig database, Table S4: SAS2A viral-like contigs similar to soil viral metagenomes from a permafrost thaw gradient.
