# Supplementary material for: Seasonal Regime Shift in the Viral Communities of a Permafrost Thaw Lake
Source: Viruses. 2020 Oct 22;12(11):1204. doi: 10.3390/v12111204 (PMC7690404; doi:10.3390/v12111204)
Supplement: Supplementary file 1 [file SuppFig S3.pdf › SuppFig S3.pdf]

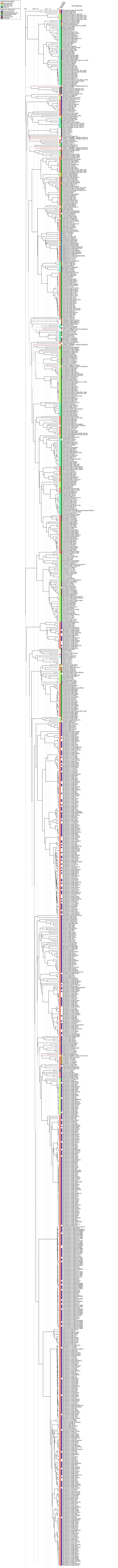

Left line: Virus family
Siphoviridae (873)
Myoviridae (163)
Podoviridae (134)
Others (7)
Right line: Host group
Actinobacteria (554)
Gammaproteobacteria (347)
Alphaproteobacteria (39)
Firmicutes (26)
Cyanobacteria (8)
Others (86)
0.001
0.005
0.01
0.05
0.1
0.5
Virus family
Host group
1210 sequences
Microbacterium phage vB_MoxS-ISF9
Clavibacter phage CN1A
Clavibacter phage CMP1
Pseudomonas phage vB_PsyM_KIL1
Pseudomonas phage vB_PaeM_PAO1_Ab17
Pseudomonas phage vB_PaeM_PAO1_Ab04
Pseudomonas phage vB_PaeM_PAO1_Ab11
Pseudomonas phage vB_PaeM_PAO1_Ab06
Synechococcus phage S-CBP42
Synechococcus phage S-CBP2
Pelagibacter phage HTVC019P
Rhizobium phage RHEph01
Mesorhizobium phage vB_MloP_Lo5R7ANS
Ralstonia phage RSB2
Delftia phage IME-DE1
Klebsiella phage KP32
Klebsiella phage K11
Pseudomonas phage PPPL-1
Pseudomonas phage phiPSA2
Pseudomonad phage gh-1
Pseudomonas phage PPpW-4
Pseudomonas phage phi15
Pseudomonas phage phiIBB-PF7A
Pseudomonas phage Phi-S1
Pseudomonas phage Pf-10
Aeromonas phage phiAS7
Xylella phage Prado
Xylella phage Paz
Pseudomonas phage Bf7
Pseudomonas phage Andromeda
Caulobacter phage Percy
Burkholderia phage JG068
Ralstonia phage RSJ5
Ralstonia phage RSJ2
Ralstonia phage RSB1
Burkholderia phage Bp-AMP2
Burkholderia phage Bp-AMP3
Burkholderia phage Bp-AMP4
Burkholderia phage Bp-AMP1
Pseudomonas phage YMC11/06/C171_PPU_BP
Acinetobacter phage Abp1
Acinetobacter phage AB3
Ralstonia phage RSB3
Pseudomonas phage phi-2
Pseudomonas phage LKA1
Pseudomonas phage LKD16
Pseudomonas phage phikF77
Pseudomonas phage vB_PaeP_PAO1_Ab05
Pseudomonas phage vB_PaeP_PPA-ABTNL
Pseudomonas phage MPK7
Pseudomonas phage DL62
Pseudomonas phage MPK6
Pseudomonas phage LUZ19
Pseudomonas phage PT5
Pseudomonas phage vB_PaeP_PAO1_1-15pyo
Pseudomonas phage vB_Pae-TbilisiM32
Pseudomonas phage phiKMV
Pseudomonas phage PT2
Pseudomonas phage MBL
VIRSorter_Ga0256681_10539383-c [83,527 nt]
Natrinema virus SNJ1
Haloarcula hispanica icosahedral virus 2
Haloarcula californiae icosahedral virus 1
Haloarcula hispanica virus SH1
Haloarcula hispanica virus PH1
Mycobacterium phage ADLER F1725
Mycobacterium phage Adler
Gordonia phage GMA6
Arthrobacter phage vB_ArtM-ArV1
Arthrobacter phage BarretLemon
Gordonia phage Orchid
Rhodococcus phage ReqiDocB7
Gordonia phage Kvothe
Gordonia phage Demosthenes
Tsukamurella phage TIN3
Tsukamurella phage TIN2
Gordonia phage Woes
Gordonia phage Monty
Gordonia phage Hotorobo
Thermus virus P74-26
Streptomyces phage Jay2Jay
Achromobacter phage JWF
