# Supplementary material for: Seasonal Regime Shift in the Viral Communities of a Permafrost Thaw Lake
Source: Viruses. 2020 Oct 22;12(11):1204. doi: 10.3390/v12111204 (PMC7690404; doi:10.3390/v12111204)
Supplement: Supplementary file 1 [file viruses-12-01204-s001.zip › supplementary material.pdf]

Supplementary Material

Seasonal Regime Shift in the Viral Communities of a Permafrost Thaw Lake

Catherine Girard [1,2,3,4,†], Valérie Langlois [1,2,3,4], Adrien Vigneron [2,3,4,5], Warwick F. Vincent [2,3,4,5] and Alexander I. Culley [1,2,3,4,*]

[1] Département de biochimie, de microbiologie et de bio-informatique, Université Laval, Québec, QC G1V 0A6, Canada.
[2] Centre d'études nordiques (CEN), Université Laval, Québec, QC G1V 0A6, Canada
[3] Institut de biologie intégrative et des systèmes (IBIS), Université Laval, Québec, QC G1V 0A6, Canada
[4] Takuvik Joint International Laboratory, Université Laval, Québec, QC G1V 0A6, Canada
[5] Département de biologie, Université Laval, Québec, QC G1V 0A6, Canada
* Correspondence: alexander.culley@bcm.ulaval.ca
† Current affiliation: Département des sciences fondamentales, Centre d'études nordiques, Université du Québec à Chicoutimi, Chicoutimi, QC G7H 2B1, Canada

Supplementary Tables

Supplementary Table S1. Processing steps from raw sequences to vOTUs. *VirSorter and VirFinder had 125 contigs in common (34.8% of total viral contigs).

Processing step	Number	Length range (median) (bp)
Paired reads	244,015,048	150
Contigs assembled	2,703,036	200 – 183,636 (404)
Viral contigs		
- VirSorter contigs >10kb		
o Category 1	21	10,115 – 36,718 (16,425)
o Category 2	179	10,022 – 80,867 (19,000)
o Category 4	2	12,576 – 83,527 (48,052)
o Category 5	7	10,785 – 29,945 (14,176)
- VirFinder contigs >2kb	275	10,057 – 80,867 (15,624)
- Total contigs*	359	10,022 – 83,527
Viral taxa (consensus sequences)	351	

Supplementary Table S2. Nonpareil viral sequence coverage estimates per sample, computed with the *k*-mer method. Kappa is the Nonpareil "Redundancy" value of the sample, C is the average coverage, LR is the actual sequencing effort (bp), ModelR is the Pearson's coefficient between subsampled data and the projected model, LRstar is the estimated sequencing effort to reach the average coverage, and diversity is the Nonpareil sequence-diversity index.

Sample	Kappa	C	LR (bp)	ModelR	LRstar (bp)	Diversity (log-bp)
B1	0.93736	0.9425819	262265299	0.9995373	229318602	15.99327
B2	0.95083	0.9549561	416579161	0.9997296	243052190	16.0623
B3	0.93104	0.9367708	285704227	0.9990797	303537185	16.01611
W1	0.82958	0.8429984	20110774	0.9997458	74235940	14.43113
W2	0.85324	0.8649497	21632203	0.9994799	66492486	14.3605
W3	0.85449	0.8661079	19966866	0.9997031	53059732	14.30925

Supplementary Table S3. vOTUs and viral-like contigs from SAS2A that had >90% identity over 75% of the shortest sequence when BLASTed against a viral contig database published by Paez-Espino et al. 2017.

See file SupplementaryTableS3.xlsx

Supplementary Table S4. Viral-like contigs from SAS2A that had >90% identity over 75% of the shortest sequence when BLASTed against a viral contig database published by Emerson et al. 2018.

See file SupplementaryTableS4.xlsx

Supplementary Figures

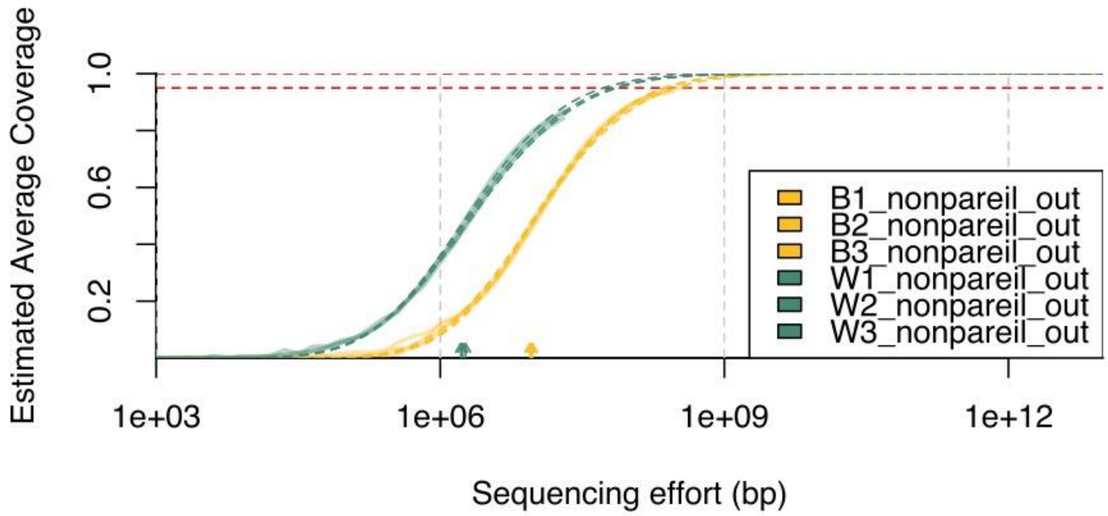

Supplementary Figure S1. Nonpareil coverage estimate curves for each replicate sampled computed using the *k*-mer method. Dotted lines show Nonpareil projection estimate curves, solid lines show coverage estimates from subsampling. Arrows show sequence diversity. The horizontal red dashed lines show 95 and 100 % coverage. Full data for Nonpareil estimates are presented in Supplementary Table S2.

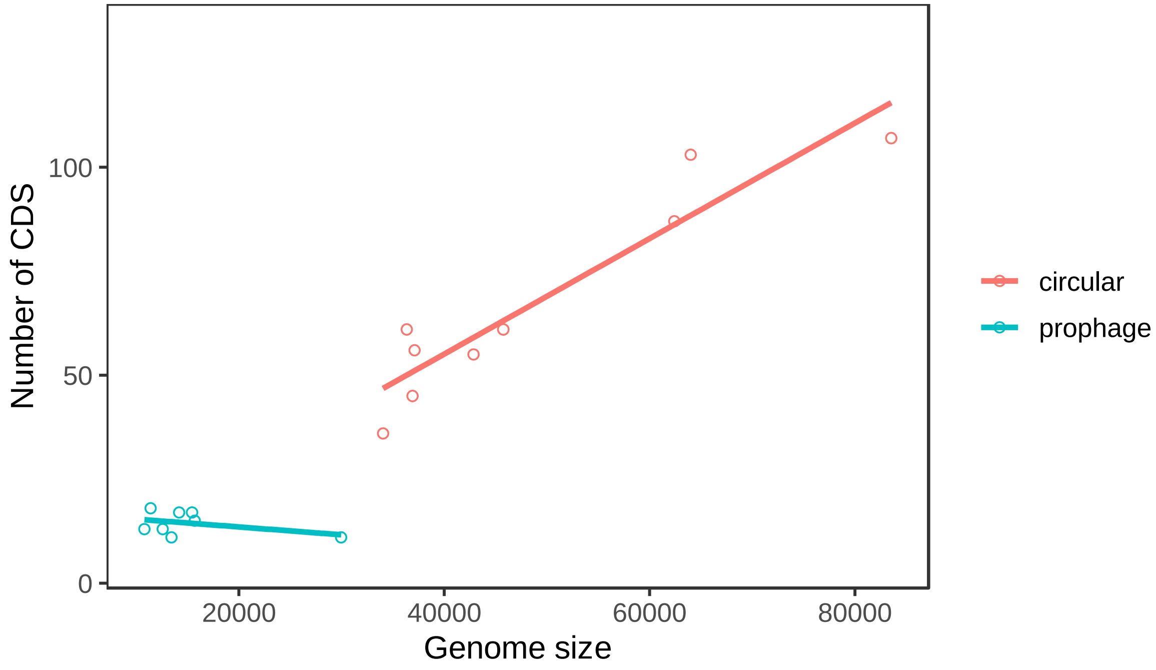

Supplementary Figure S2. Linear regression models between genome length and number of CDS (predicted by Prokka) in circular (red) and prophage (blue) genomes. The line shows the significant linear relationship within the circular vOTU dataset (adjusted R^2 = 86%, P = 0.0001). The plot for the prophage dataset is also shown. The relationship was not significant.

Supplementary Figure S3. Viral proteomic tree plotted with ViPTree with 1201 reference sequences and the 9 circular UViGs from this study.

See file SuppFigureS3.pdf
